# Acute relaxation during pregnancy leads to a reduction in maternal electrodermal activity and self-reported stress levels

**DOI:** 10.1186/s12884-021-04099-4

**Published:** 2021-09-17

**Authors:** Ilena Bauer, Julia Hartkopf, Anna-Karin Wikström, Nora K. Schaal, Hubert Preissl, Birgit Derntl, Franziska Schleger

**Affiliations:** 1 German Center for Diabetes Research (DZD e.V.), fMEG-Center, Institute for Diabetes Research and Metabolic Diseases of the Helmholtz Center Munich/ fMEG Center, University of Tuebingen, Otfried-Müller-Strasse 47, 72076 Tuebingen, Germany; 2 Department of Women’s and Children’s health, Uppsala University, Uppsala, Sweden; 3 Department of Experimental Psychology, Heinrich-Heine-University, Duesseldorf, Germany; 4 Department of Internal Medicine IV, Division of Endocrinology, Diabetology, and Nephrology, University Hospital Tuebingen, Tuebingen, Germany; 5 Department for Psychiatry and Psychotherapy, Tuebingen Center for Mental Health (TüCMH), University of Tuebingen, Tuebingen, Germany; 6 LEAD Graduate School & Research Network, University of Tuebingen, Tuebingen, Germany

**Keywords:** Pregnancy, Relaxation, Maternal stress, Heart rate, Electrodermal activity

## Abstract

**Background:**

Prenatal maternal stress can have adverse effects on birth outcomes and fetal development. Relaxation techniques have been examined as potential countermeasures. This study investigates different relaxation techniques and their effect on self-reported stress levels and physiological stress levels in pregnant women.

**Methods:**

In this cross-sectional study, 38 pregnant women in their 30th to 40th gestational week were assigned to one of three, 20-min lasting relaxation groups: listening to music (*N* = 12), following a guided imagery (N = 12) or resting (N = 12). The intervention, i.e., acute relaxation (music, guided imagery or resting) took place once for each study participant. Study inclusion criteria were age over 18 years, German speaking, singleton and uncomplicated pregnancy during the 30th and 40th week of gestation. The stress levels were determined during the study. Current stress level during the study was assessed by a visual analogue scale. Chronic stress levels were assessed by the Trier Inventory of Chronic Stress and the Pregnancy Distress questionnaire. Multivariate analyses of covariance were performed and dependent measures included stress levels as well as physiological measures, i.e., cardiovascular activity (electrocardiogram) and skin conductance levels.

**Results:**

All three forms of relaxation led to reduced maternal stress which manifested itself in significantly decreased skin conductance, F(3,94) = 18.011, *p* = .001, η_p_^2^ = .365, and subjective stress levels after the interventions with no significant group difference. Post-intervention stress ratings were further affected by gestational age, with less subjective relaxation in women later in gestation, F (1, 34)=4.971, *p* = .032, η_p_^2^ = .128.

**Conclusion:**

Independent of relaxation technique, single, 20-min relaxation intervention (music, guided imagery or resting) can significantly reduce maternal stress. Notably, women at an earlier stage in their pregnancy reported higher relaxation after the intervention than women later in gestation. Hence, gestational age may influence perceived stress levels and should be considered when evaluating relaxation or stress management interventions during pregnancy.

**Trial registration:**

Not applicable.

**Supplementary Information:**

The online version contains supplementary material available at 10.1186/s12884-021-04099-4.

## Background

Pregnancy is characterized by various changes, including hormonal changes, different personal expectations and a new coordination of the professional and social environment, often accompanied by financial or health concerns [1–3]. Depending on the circumstances, the degree of support from the environment and many other (individual) factors, these changes can lead to intense emotional states and stress [4]. A negative maternal emotional state or maternal stress over a longer period of time not only affects mental health of the becoming mother but can interfere with the development of the offspring: Several studies have shown adverse effects of intense negative maternal emotional states, ranging from prenatal distress to peripartum mental disorders, on fetal and infant development [5–8]. More specifically, adverse effects on the physiological, metabolic and neuronal development of the fetus have been reported [9–11]. The fetal autonomic nervous system reacts and adapts rapidly to environmental changes: Higher fetal heart rate variability and lower fetal heart rate are indicators for fetal well-being. Accordingly, several studies have shown that increased maternal depressive or stress symptoms lead to changes in fetal cardiovascular activity [12–14]. Furthermore, recent studies reported a relation between maternal stress and the offspring’s physiological stress reactivity and cortisol levels [15–18]. Altered maternal cortisol levels due to high and chronic stress have been hypothesized to be related to the development of emotional and behavioral psychopathology across the child’s lifespan, potentially also affecting cognitive performance and brain volume [19–21]. In addition, maternal diurnal cortisol was related to the newborn’s stress reactivity and thus to the development of the hypothalamic-pituitary-adrenal axis, as measured by a neonatal heel-stick measurement [22].

To avoid or compensate possible adverse effects caused by maternal stress, preventive measures such as relaxation techniques have become increasingly important. So far, most studies indicate that maternal relaxation during pregnancy can reduce maternal stress and improve maternal wellbeing [23, 24]. In pregnant women, a significant number of studies have shown that relaxation strategies not only positively influence the maternal autonomic nervous system but also reduce symptoms of maternal anxiety and depression [25–29]. For instance, DiPietro and colleagues [28] administered 18-min of guided imagery and music for relaxation during the third trimester of pregnancy. Significant changes in maternal heart rate (HR) and skin conductance level (SCL) were shown. A questionnaire-based study by Nwebube and colleagues [26] detected lower anxiety and depressive symptoms in pregnant women receiving relaxation music for twelve weeks of their pregnancy compared to a control group. Following a prenatal music intervention for relaxation, a significant decrease in maternal systolic and diastolic blood pressure, heart rate [30] and uterine contractions [31] was reported. Moreover, relaxation may have a positive effect on the perception of maternal pain during labor [32]. Over the last decade, several studies have reported significant effects of different relaxation types on fetal and neonatal development [33, 34]. The study by DiPietro and colleagues [28], for example, reported a reduced fetal heart rate and increased fetal heart rate variability. In summary, a positive influence of different relaxation techniques on mothers’ well-being as well as fetal and neonatal development, can be assumed.

Previous studies investigating the influence of relaxation techniques on maternal and fetal well-being used different types of relaxation methods (active/passive, body-based, mental-based) with different duration and frequency (regular, single, weekly). Since physical relaxation techniques may not be suitable for every woman during pregnancy, the need for evidence-based mental techniques that can be used regardless of a woman’s physical condition has increased. To our knowledge, none of the previous studies directly compared different mental-based active/passive relaxation interventions in pregnant women. We categorized guided imagery as active relaxation on the basis of the instructions given and listening to music as a passive form of relaxation. Therefore, the present study focuses on the question which type of mental-based active or passive relaxation technique leads to greater relaxation during pregnancy.

Three commonly used mental-based relaxation forms, music (listening to music), a guided imagery (following a guided imagery without body-based instructions) and minimal relaxation (resting, i.e., sitting quietly), are compared with regard to their effectiveness on maternal physiological, and psychological parameters of stress during the third trimester of pregnancy. In our study, guided imagery was categorized as active relaxation on the basis of the instructions given while listening to music was categorized as a passive form of relaxation.

The following hypotheses were defined:

(1) We anticipated that acute relaxation, with either music, guided imagery or resting, leads to a decrease in physiological stress levels, i.e., decreased maternal heart rate and SCL.

(2) We expected the effects of the three interventions to differ, with a significantly stronger effect on physiological parameters during the relaxation for guided imagery (active relaxation) than music or during resting (passive relaxation).

(3) The subjective effects of the three relaxation conditions, which were also of interest to us, were assumed to decrease after the intervention.

In an exploratory analysis, we also investigated whether gestational age (GA) influenced any of the stress ratings assessed.

## Methods

This cross-sectional study was conducted between February 2018 and September 2019. The Ethics committee of the Medical Faculty of the University of Tuebingen, Germany, approved the study (748/2017BO1). Informed consent of all participants was obtained prior to the start of the measurements.

### Participants

Pregnant healthy women were recruited by electronic communication. Eligibility was restricted to women over 18 years of age, German speaking, singleton, uncomplicated pregnancy during the 30th and 40th week of gestation. Exclusion criteria were hearing impairments, diagnosed mental disorders (self-report) or drug/nicotine consumption during pregnancy (self-report). Once initial information about the study had been provided, a single visit was scheduled between 30th and 40th week of gestation. A total of 38 women were enrolled. Three out of 38 women stated that they were vegetarian or vegan and one woman stated that she had celiac disease. With regard to any previous experience with relaxation, four women reported that they had not previously used strict routine activities to encourage relaxation. Of those who did, the most commonly reported techniques were yoga and meditation, exercising, daytime naps or reading. Three women stated that they also liked to listen to music to help them relax. On average, participants were 30.9 years old, in their 34th week of gestation, and 72% were expecting their first child. Nine out of 38 women participated in another measurement in our center only shortly before the reported study. These participants had rested for 30 min but without any specific intervention. Two-thirds of the women had a college or a higher education/university degree. Participants were informed about the course of the study and gave their written informed consent prior to participation.

### Design and procedure

The participants were alternately assigned to the *music group*, the *guided imagery group* or the *resting group* shortly before the measurement commenced. We scheduled a 1 h-visit between 8 am and 2 pm. On each participant’s arrival, the in-house midwife checked fetal vitality with a pinard horn. All women were asked to fill out a questionnaire (Profile of Mood States, POMS) to assess their current mood [35]. They also rated their current stress level on a visual analogue scale (VAS) ranging from 0 (not stressed at all) to 10 (highly stressed). The women filled out both paper-pencil questionnaires at our center before and after the intervention.

The relaxation intervention took place for each woman individually and personally. It started once the woman had been positioned in a semi-recumbent comfortable armchair in a noise-reduced room. During the intervention, the light in the room was dimmed. The electrodermal activity- and heart rate monitor was positioned out of view of the participants. Women were asked whether the room temperature was comfortable, or whether or not they were cold and whether they felt generally comfortable. In all three groups, we began with a test measurement for three minutes to ensure that the electrodes were in working order. Afterwards, ten minutes of baseline data were collected, during which we gave instructions to remain quietly seated, breathe calmly and avoid any movements. Subsequently, headphones were given to the women (also in the *resting group* to ensure comparability between conditions) and the intervention was initiated (only for *music group* and *guided imagery group*) for the next 10 min. The music (*“Find Your Inner Peace”,* Rostar) was designed specifically for relaxation, using certain tempos for inducing a calm state but no vocals. The guided imagery text was designed for use during pregnancy by a midwife on the basis of her own professional experience and adapted specifically for this study. To avoid conscious active physical tension, which might cause artifacts in the data, the guided-imagery contained no body-related instructions. For the resting condition, the women were requested to remain seated quietly without moving and to breathe normally. Following the relaxation period, a 10-min interval served as a recovery measurement (i.e., no specific relaxation intervention). During the entire procedure, our in-house midwife and the study assistant remained in the room with the woman. Once the measurement was complete, the study assistant removed the electrodes and headphones.

**Fig. 1:**
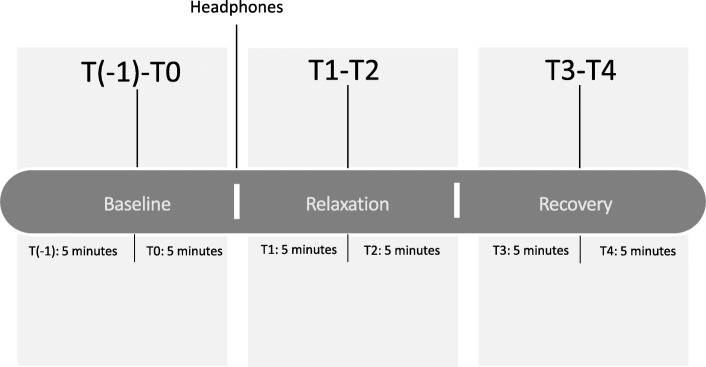
Study design. The measurement procedure was divided into three phases; baseline, relaxation, and recovery measurement. Headphones were provided at the beginning of T1 in all groups. Each phase was divided into five-minute segments for analysis. All three groups (music, guided imagery, resting) completed the same periods (Baseline, Relaxation, Recovery) of the study

Afterwards, all women rated their stress level again on the POMS and VAS and indicated how they had experienced the intervention. After their participation, they received a link for additional questionnaires via email assessing chronic stress and pregnancy-related distress (see details below). They completed these questionnaires at home, one to 5 days after their appointment at our center. We used the software Unipark (www.unipark.de) for the computer-based questionnaires. Since it has turned out that putting on headphones causes an interruption in our data, for data analysis we only took into account those segments after the beginning of the intervention (relaxation) phase (T1) until the end of the recovery phase (T4; see Fig. 1). The total time of the relaxation phase and the recovery phase was thus 20 min. For the analysis of the maternal physiological activity, we used baseline correction by subtracting the mean of the first five minutes at the beginning (T0) without any intervention from the data measured during the period from T1 to T4.

Testing was discontinued for two participants due to technical issues and discomfort of the women. The data analysis is therefore based on the remaining 36 participants. We included the following questionnaires for the analysis:

#### Prenatal distress questionnaire (PDQ) – Pregnancy-related distress

The Prenatal Distress Questionnaire (PDQ) uses 12 items to assess pregnancy-related and birth related concerns [36]. The questionnaire includes five response categories (from 0 = never to 4 = always). PDQ scores ranged from 0 to 48. The Cronbach’s alpha for the overall PDQ score was consistently reported to lie between 0.80 and 0.81 [37] and test–retest reliability has been reported to be r = 0.75 [38]. The PDQ has been found to have good convergent validity, since it is significantly correlated with general stress measures (State Trait Anxiety Inventory – State Scale, Life Event Stress and Perceived Stress Scale) [37].

#### Trier inventory of chronic stress (TICS) – chronic stress

For the assessment of chronic stress, we used the Trier Inventory of Chronic Stress (TICS) questionnaire [39]. This questionnaire is based on an interaction-related stress concept [40] according to which stress arises in and through the active confrontation of a person with the demands of their environment. The questionnaire includes 57 items form 9 different subscales: Work overload, social overload, pressure to succeed, dissatisfaction with work, excessive demands at work, lack of social recognition, social tensions, social isolation and chronic concerns. Participants answered using a 5-point Likert scale response format (0 = never to 4 = always). In addition, its screening scale for chronic stress (SSCS), which uses 12 items to record chronic stress in a non-specific and global manner, was used for the analysis. An evaluation period of the last 3 months is required. The internal consistencies (Cronbach’s alpha) of the scales range from .84 to .91 (M = .87). The Rasch reliabilities range from .78 to .89 (M = .83). The procedure has good profile reliability (.72). Numerous results on construct validity (factor analyses, correlations with stress questionnaires, personality traits, partnership behavior, social support, sleep quality, physical and psychological complaints, cortisol release) are available for the TICS. Furthermore, the TICS profiles of different study groups endorse for the validity of the procedure [39].

#### Profile of mood states (POMS)- current mood

In the present study, the German short form of the POMS with 35 items and 7-point Likert scale response format (0 = not at all to 6 = very strong) was used [35]. The 35 items form the scales of depression/anxiety, fatigue, vigor and hostility. In the POMS, the participants were asked to evaluate their state of mood over the last 24 h. The POMS appears to be an internally consistent instrument and the Cronbach’s Alpha ranged from 0.89 to 0.95. There are indications of convergent validity of POMS-scales with two questions: 1. Over the past 2 weeks, have you felt down, depressed, or helpless? and 2. Over the past 2 weeks, have you felt little interest or pleasure in doing things? [35].

#### Maternal physiological response

Maternal electrodermal and cardiovascular activity were recorded with a four-channel data acquisition and analysis device (MP36R Research System, BIOPAC (USA)). Data were recorded at a sampling rate of 2000 Hz. The electrocardiogram (ECG) was recorded from three disposable electrodes (EL503, Biopac Systems, Inc., CA) which were placed on the forearms of the participants. Data quantification was processed offline using Matlab R2018a (The MathWorks, Natick, MA). ECG data underwent R-peak detection (in house software), manual editing for artifacts and inter-beat interval computation. The time-domain parameters included root mean square of successive differences (RMSSD) and the heart rate (beats per minutes (bpm)). Electrodermal activity (quantified by SCL) was measured by administering a constant 0.5 Volt root-mean square 35 Hz AC excitation signal and detecting the current flow. SCL was monitored from two disposable pre-gelled electrodes (EL507, Biopac Systems, Inc., CA). These were placed on the distal phalanxes of the index and middle finger of the non-dominant hand. Electrodes were fixed in position with adhesive tape. Before the measurement, all women took off their shoes to avoid any sudden spikes in the SCL trace due to rubber-soled shoes [41]. Women were instructed to sit quietly and reduce any movement. SCL was scaled from 0 to 25 microsiemens. Data quantification continued offline using Biopac software (*Acqknowledge5* (CA, USA)). Artifacts were substituted with linear interpolation based on the values at the left and right edges if necessary and with a median smoothing with 50 samples to delete movement artifacts [42]. All values were baseline corrected for the analysis and the complete data set was divided into 5-min sections, via which the mean value was then calculated.

## Data analysis

All statistical analyses described below were performed using the software program SPSS (*IBM SPSS Statistics 26*), alpha levels were set to *p* < .05.

Data preparation of all dependent variables included tests for normality, homogeneity of variances and examination of outliers. Where not normally distributed, variables were subjected to transformation by natural logarithm and adding a constant, or were ranked prior to the application of the statistical procedures.

Outliers were removed if values were more than three standard deviations from the mean value. Excluding these individual data points lead to a sample size of seven to twelve subjects per group (for details see Table 2). As some women were participating in another measurement before the current study and thus were probably more at ease in the clinical environment, we included this variable as covariate in all analyses. Additionally, we also included maternal chronic stress in our analyses because different chronic stress levels can have an impact on maternal baseline values.

Group differences in all dependent variables (maternal heart rate, maternal skin conductance level and subjective stress) were evaluated using mixed-effects ANCOVAs with the between-subjects factor group (*music, guided imagery, resting*) and the within-subject factor time (T1-T4) and the two covariates ‘participating in another measurement before at our center’ and ‘chronic stress’. To analyze effects of time within relaxation phase and recovery phase separately, we used a repeated measures ANCOVA (rmANCOVA). We used Bonferroni or Dunnett T3 correction for post-hoc analysis. In case of non-normal distribution of the data, Wilcoxon signed-rank test were applied.

State and trait questionnaires were analyzed by the standard evaluation procedure for every questionnaire (POMS, PDQ and TICS) and all data were analyzed using MANCOVAs with group and/or time as factors (POMS, TICS, VAS).

Before and after the measurement, we asked all participants to use a VAS to indicate how stressed they felt at that particular point in time. To analyze the VAS, we measured the distance from 0 to the set mark in cm to gain the pre (before the measurement) and post values (after the measurement).

Explorative analysis of differences in VAS between GA groups (Group 1 (30–34 GA) and Group 2 (35–40 GA)): For the mean delta value included in the analysis, we subtracted the pre-value from the post-value for each woman separately. An ANCOVA was used to determine the differences between GA groups. The dependent variable was the mean of VAS delta to describe changes in subjective stress pre vs. post intervention.

## Results

**Fig. 2:**
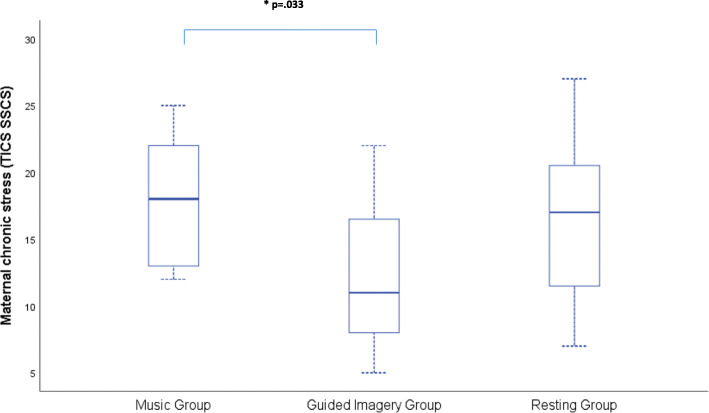
Trier Inventory of Chronic Stress (TICS)

**Fig. 3:**
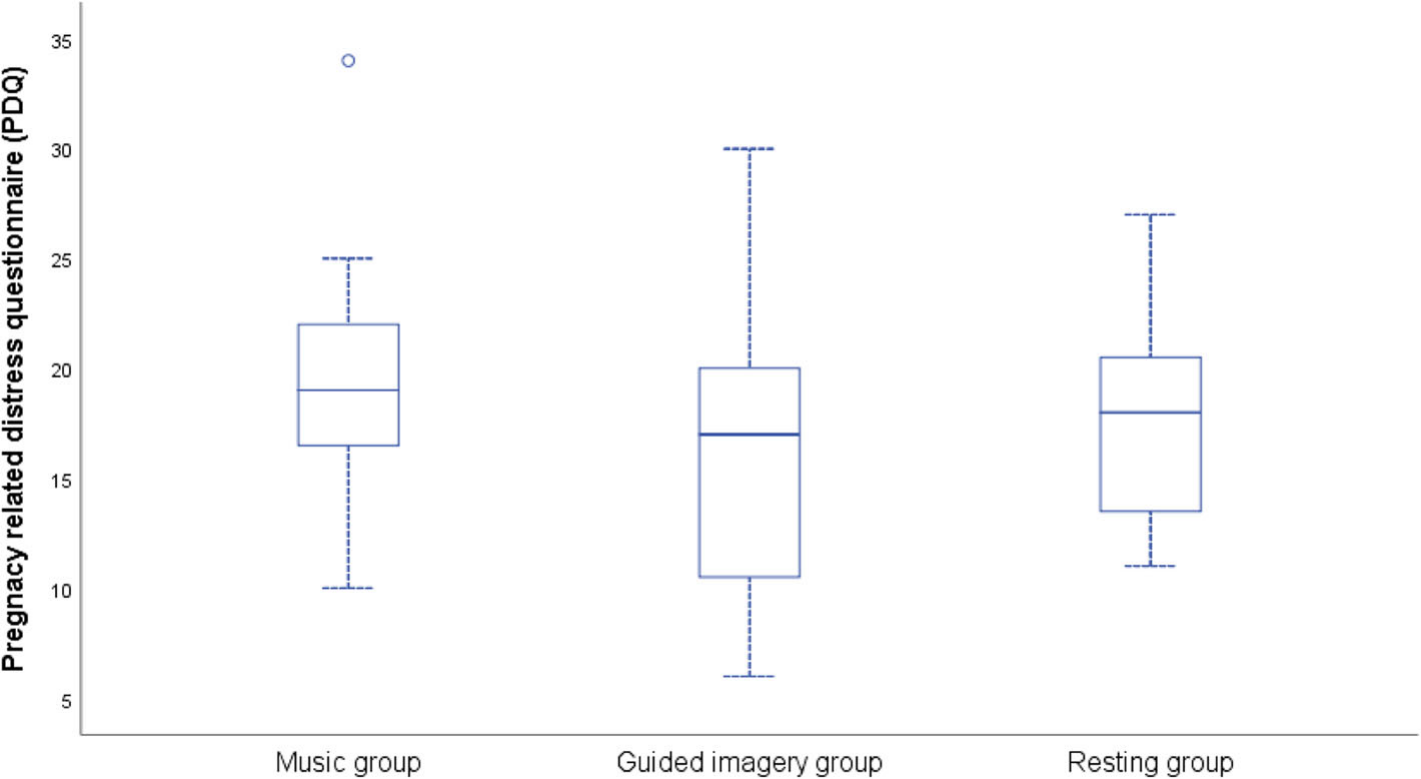
Pregnancy related distress Questionnaire (PDQ). Short Scale of Chronic Stress (SSCS): Overview of mean values in all groups: music, guided imagery, resting. Scale of pregnancy-related distress: Overview of mean values in all groups: music, guided imagery, resting

No significant differences were detected between groups in fetal characteristics (e.g. sex, for descriptions see Table 1). To determine group differences, we performed a MANOVA with factor group (*music group, guided imagery group, resting group*) and total score of TICS and PDQ, maternal age and GA. While maternal age and GA did not differ between the groups, F (2, 33)=2.424, *p* = .122; F (2, 33)=2.875, *p* = .071, the total score total of chronic stress (screening scale of chronic stress (SSCS)) and ‘participating in another measurement before’ differed significantly, F (2, 33)=3.808, *p* = .033, η_p_^2^ = .187; F (2, 33)=6.556, *p* = .004, η_p_^2^ = .291. Bonferroni post-hoc test revealed a significant difference in SSCS between the music group and the guided imagery group (see Fig. 2). Pregnancy-related distress (PDQ) did not differ significantly between groups, F (2, 33)=1.221, *p* = .308 (see Fig. 3). Group characteristics are presented in Table 1*.*

**Table 1:** Characteristics of participants

Group	Gestational Age (GA) (mean) in weeks (SD)	Maternal Age (mean) in years (SD)	Primiparous/Multiparous
All groups (total)	33.56 (3.21)	30.86 (4.30)	26/10
*Music Group*	34.50 (3.15)	30.08 (3.85)	11/1
*Guided Imagery Group*	31.83 (3.13)	29.58 (5.09)	8/4
*Resting Group*	34.33 (2.87)	32.92 (3.34)	7/5

Baseline characteristics of participants in the groups: music (*N* = 12), guided imagery (*N* = 12) and resting (*N* = 12) intervention and all groups in total (*N* = 36)

### Maternal cardiovascular response to relaxation procedures

Variables describing maternal cardiovascular activity are presented in Table 2.

**Table 2:** Overview of maternal cardiovascular activity

Type of intervention		HR (T1)	HR (T2)	HR (T3)	HR (T4)	RMSSD (T1)	RMSSD (T2)	RMSSD (T3)	RMSSD (T4)
**Music Group**	*Mean*	− 1.66	−.86	−2.33	− 2.48	5.19	7.42	8.70	7.50
	*Standard error*	.90	.89	.88	1.63	3.96	4.58	3.90	3.45
	*Standard deviation*	3.12	2.96	2.90	5.63	13.71	15.87	13.53	11.44
	*N*	12	11	11	12	12	12	12	11
**Guided** **Imagery Group**	*Mean*	−3.14	−3.90	−1.87	−1.40	4.67	9.48	2.61	−.39
	*Standard error*	.74	1.12	.55	.91	1.21	3.13	.77	1.57
	*Standard deviation*	2.45	3.70	1.81	3.03	4.03	10.38	2.43	5.22
	*N*	11	11	11	11	11	11	10	11
**Resting Group**	*Mean*	−1.09	−2.25	−2.44	−2.58	−1.05	6.89	1.40	9.92
	*Standard error*	.72	1.68	.97	1.24	3.16	6.83	1.95	7.49
	*Standard deviation*	2.48	5.83	3.38	4.31	9.98	23.67	6.47	25.95
	*N*	12	12	12	12	10	12	11	12
**All Groups**	*Mean*	−1.93	−2.33	−2.22	−2.17	3.13^a^	7.88	4.42	5.80
	*Standard error*	.47	.76	.47	.74	1.79	2.91	1.64	2.93
	*Standard deviation*	2.77	4.43	2.72	4.39	10.27	17.20	9.42	17.10
	*N*	35	34	34	35	33	35	33	34

All values are baseline corrected. Maternal Heart Rate (HR), Root Mean Square of Successive Differences (RMSSD). T1-T2: Relaxation phase; T3-T4: Recovery phase

#### Maternal heart rate

A MANCOVA with factors group (*music, guided imagery, resting)* and time (T1 to T4) revealed no significant main effects for time, F(3,94) = 0.109, *p* = .955, or group, F(2,94) = .317, *p* = .729, and no significant interaction between group and time, F(6,94) = 1.007, *p* = .426.

#### Maternal heart rate variability

For heart rate variability, we used the root mean square of successive differences (RMSSD). A MANCOVA with factors group (*music, guided imagery, resting*) and time (T1 to T4) revealed no significant main effects of time, F(3,94) = 2.143, *p* = .100, or group, F(2,94) = 0.624, *p* = .538, and no significant interaction between group and time, F(6,94) = 1.339, *p* = .248.

##### Relaxation vs. recovery phase

To further determine whether a relaxation effect sets in during the recovery phase, we analyzed whether there is a difference between the relaxation (delta of mean T1-T2) and the recovery (delta of mean T3-T4) phase in maternal parameters.

A MANCOVA with factors group (*music, guided imagery, resting*) and change of time (delta) revealed neither a significant main effect of group, F(2,56) = 0.824, *p* = .444, nor time, F(1,56) = 2.181, *p* = .145, or group-by-time interaction, F(2,56) = .626, *p* = .538, for maternal heart rate. Similarly no significant main effect of group, F(2,56) = .377, *p* = .688, or time, F(1,56) = .815, *p* = .371, or group-by-time interaction, F(2,56) = .245, *p* = .784, was found for maternal RMSSD.

#### Maternal electrodermal response to relaxation procedures

We assumed a significant relaxation effect over time within each group, with decreasing SCL values, and differences in the size of this effect between groups in maternal SCL. Maternal SCL data is presented in Table 3.

**Table 3:** Overview of maternal electrodermal activity

Overview of maternal SCLs					
**Type of intervention**		**SCL (T1)**	**SCL (T2)**	**SCL (T3)**	**SCL (T4)**
**Music Group**	*Mean*	1.03	.12	.01	−.04
	*Standard error*	.26	.07	.04	.05
	*Standard deviation*	.89	.21	.14	.17
	*N*	12	10	10	10
**Guided Imagery Group**	*Mean*	1.57	−.22	.20	−.72
	*Standard error*	.41	.26	.24	.46
	*Standard deviation*	1.28	.83	.73	1.46
	*N*	10	10	9	10
**Resting Group**	*Mean*	1.32	.12	−.08	−.14
	*Standard error*	.39	.18	.13	.17
	*Standard deviation*	1.36	.61	.45	.58
	*N*	12	12	12	12
**All Groups**	*Mean*	1.29	.01	.03	−.29
	*Standard error*	.20	.11	.09	.16
	*Standard deviation*	1.17	.61	.49	.92
	*N*	34	32	31	32

A MANCOVA with factors group (*music, guided imagery, resting*) and time (T1 to T4) revealed a significant main effect of time, F(3,94) = 18.011, *p* = .001, η_p_^2^ = .365, but no group effect, F(2,94) = .075, *p* = .928. The interaction between group and time was not significant, F(6,94) = 1.192, *p* = .317. For secondary analysis and to determine time effect for each group separately, a repeated measures ANCOVA with a Greenhouse-Geisser correction revealed no significant main effects of time within the *music group*, F (3, 21)=1.250, *p* = .301, the *guided imagery group*, F (3, 24)=.122, *p* = .836, and the *resting group*, F (3, 30)=0.152, *p* = .771, separately. Hence, no significant relaxation effect over time could be determined.

##### Relaxation vs. recovery phase

A MANCOVA with factors group (*music, guided imagery, resting*) and change over time (delta) revealed no significant main effect of group, F(2,56) = 2.856, *p* = .066, but a significant main effect of time, F(1,56) = 21.935, *p* = .001, η_p_^2^ = .281, in maternal SCL. The interaction between group and time was not significant, F(2,56) = .490,*p* = .615. A Wilcoxon signed-rank test revealed a significant difference between relaxation and recovery phase in SCL within every group. For women in the *music group*, the relaxation effect decreased significantly in the recovery phase compared to the relaxation phase, z = − 2.666, *p* = .008, r = 0.61, same for women in the *guided imagery group*, z = − 2.191, *p* = .028, r = 0.58, and *resting group*, z = − 2.981, *p* = .003, r = 0.61. To explore potential group difference in the long-term impact of the relaxation intervention during the recovery phase, delta SCL values of the recovery phase only, were subjected to a MANCOVA with the factor group (*music, guided imagery, resting*). The analysis revealed no significant difference between the groups, F (2, 27)=1.700, *p* = .202. The values in the recovery phase therefore did not differ significantly between interventions.

### Maternal subjective response to relaxation procedures

On the basis of our third hypothesis, we assumed that there would be a decrease in stress levels in all groups independent of the type of relaxation administered. The MANCOVA with group as between-subject factor (*music, guided imagery, resting*) showed no significant main effect of group on delta of mean levels of the VAS for subjective stress (Post-Pre), F (2, 31)=.583, *p* = .564.

Albeit we did not observe a significant group effect on VAS levels, a Wilcoxon signed-rank test determined a significant time effect within groups for the VAS (pre vs. post). Maternal stress levels were significantly reduced in the *music group*, z = − 2.936, *p* = .003, r = 0.84, in the *guided imagery group*, z = − 2.934, p = .003, r = 0.84, and in the *resting group*, z = − 3.059, *p* = .002, r = 0.88.

A MANCOVA with group as between-subject factor *(music, guided imagery, resting)* showed no significant effect in delta of mean POMS (Pre-Post) levels for each subscale, F (2, 31)=.952, *p* = .397 (depression/anxiety), F (2, 31)=2.366, *p* = .111 (fatigue), F (2, 31)=2.222, *p* = .125 (vigor), F (2, 31)=1.831, *p* = .177 (hostility).

With regard to the POMS, a rmANCOVA with group as factor showed no significant interaction effect for all four subscales, depression/anxiety: F (1, 33)=2.178, *p* = .129; fatigue: F (1, 33)=1.826, *p* = .177; vigor: F (1, 33)=1.074, *p* = .353 and hostility: F (1, 33)=2.035, *p* = .147. However, we found significant main effects of time for depression/anxiety, fatigue and hostility in each group. In particular, the improvement in depression/anxiety was shown for the *resting* and *music group* but not for the *guided imagery group*. For statistical details, see Table 4.

**Table 4:** Overview of subscales of Profile of Mood States (POMS)

Paired t-test	**POMS Subscales**	*Music Group*	*Guided Imagery Group*	*Resting Group*
	*Depression/anxiety*	t (11)=2.698, *p* = .021*, d_z_ = 0.73	t (11)=2.007, *p* = .070	t (11)=4.201, **p = .001**, d_z_ = 1.21
	*Hostility*	t (11)=1.605, *p* = .137	t (11)=2.166, *p* = .053	t (11)=3.895, *p* = .**002**, d_z_ = 1.12
	*Fatigue*	t (11)=2.538, *p* = .028*, d_z_ = 0.73	t (11)= − 0.192, *p* = .851	t (11)=0.967, *p* = .354
	*Vigor*	t (11)=1.575, *p* = .143	t (11)=3.178, *p* = .009*, d_z_ = 0.91	t (11)=2.726, *p* = .020*, d_z_ = 0.79

*on a statistical level of*p* **< 0.05 significant**

In addition, we were interested in whether the effects we found for the VAS were dependent of GA:

In an exploratory approach, we divided women into two groups on the basis of their GA (Group 1: 30th–34th gestational week, *N* = 21; Group 2: 35th–40th gestational week, *N* = 15). The difference in the pre-rating values of VAS between the groups was not statistically significant, F (1, 34)=1.318, *p* = .259. Numerically, women with higher GA (Group 2) had lower values in the pre-rating, meaning that they might have been somewhat less stressed (Mean (SD) Group 1: 2.33 (1.59); Group 2: 1.79 (1.72)) beforehand.

As an explorative approach, an ANOVA showed a significant difference between GA groups in mean VAS delta, F (1, 34)=4.971, *p* = .032, η_p_^2^ = .128, i.e., women with higher GA had less change in VAS levels (delta mean: 1.02) compared to women with lower GA (delta mean: 1.23). Thus, women earlier in the third trimester (30th–34th gestational week) appear to be able to relax more easily compared to women with higher GA. However, when we included chronic stress and ‘participating in another measurement before at our center’ as covariates, this effect was no longer significant, F (1, 32)=2.113, *p* = .156.

## Discussion

This study aimed to investigate the effect of three different types of relaxation interventions on subjective and physiological stress-related parameters in the last trimester of pregnancy. We observed that maternal physiological measures (HR, SCL) showed a decrease independent of the type of intervention, albeit not to a significant extent.

Notably, we observed a significant difference for all groups in the change of SCL between the relaxation and the recovery phase independent of intervention. Thus, relaxation effects are still ongoing during the recovery phase, but are less intense than in the relaxation phase. Furthermore, our study shows a significant improvement in women’s subjective stress levels and mood independent of intervention, with slight differences in subscales between interventions.

In our study GA was associated with subjective stress ratings, indicating that women in later stages of pregnancy (35th–40th gestational week) did profit less from relaxation than women in the earlier weeks of gestation (30th–34th gestational week).

However, we did not find any significant differences between active (*guided imagery group*) and passive (*music group, resting group*) mental-based interventions.

The fact that no significant differences were observed between the interventions indicates that quiet, comfortable sitting can, in certain situations, be just as effective as an active mental-based relaxation technique in the short term, on both objective and subjective relaxation parameters. Moreover, the significant differences between relaxation and recovery phase emphasize that duration of relaxation might play a role. We showed that, during a 20-min relaxation and recovery period, the relaxation effect increased over time. These results are consistent with previous studies which described an ongoing decrease in maternal physiological parameters following relaxation interventions in pregnant women [30, 43].

In our study, we used different relaxation techniques, but explicitly considered each technique separately to determine possible differences in effectiveness between acute active and acute passive relaxation techniques. For maternal heart rate, we found an increase for the *guided imagery group* despite the fact that women rated the guided imagery as relaxing. This may be due to the active relaxation stimulation. However, when comparing all participants, we did not find significant differences between the groups. This is in line with a study by Teixera and colleagues [25] who showed a greater general decrease in maternal heart rate following combined active and passive relaxation for 58 women between 28th and -34th weeks of gestation. A study by Urech and colleagues [44] reported significant differences between mental-based active (guided imagery), body-based active (progressive muscle relaxation) and passive (quiet sitting) relaxation intervention in 39 healthy pregnant women on the basis of subjective ratings and cardiovascular activity.

To our knowledge, this study is the first of its kind showing a significant decrease in SCL from relaxation to recovery after a short acute relaxation intervention in pregnant women. The direction of this effect is not in line with a study by DiPietro and colleagues [28], who reported a difference in SCL between the relaxation and recovery phase, showing an increase in the latter. However, in their study a combined relaxation intervention of progressive muscle relaxation, audio-recorded guided imagery and self-selected music was used. The baseline measurement, the following stimulation intervention and the post-relaxation phase each lasted 18 min. In addition, 41% of the participants were given a 18-min pre-baseline (rest). It should be noted that in the study of DiPietro and colleagues, relaxation and recovery was interrupted when the lights were turned on and different questions were answered. This might explain why they reported a significant increase in SCL from baseline to relaxation and from relaxation to recovery. Additionally, DiPietro and colleagues showed positive effects on subscales such as physiological tension, physical assessment and cognitive tension which are in line with our results.

In general, our results show that an acute relaxation intervention during pregnancy without disturbance for 10 min can still lead to relaxation when followed by a recovery phase (also lasting for 10 min). All types of interventions used were effective in generating a subjective feeling of relaxation as indicated by a significant decrease in subjective stress ratings post- compared to pre-relaxation intervention. Our results indicate overall lower subjective stress symptoms after relaxation induction and a decrease in depressive and anxiety symptoms, and are therefore in line with the literature [25, 26, 44–46].

Notably, in this study we included the maternal chronic stress level as covariate since participants in the three intervention groups had significantly different chronic stress levels, which can be a driving factor in stress perception.

As pointed out above, stress during pregnancy can have serious consequences for the mother and the child. Clinical interest in the prevention of stress and depression during and after pregnancy is therefore high. The results of this study may be a starting point in helping to find a particularly suitable intervention for pregnant women and to develop brief but effective relaxation programs for practical use.

### Limitations

There are several limitations to this study. All participating women were at our center for the first time, but some women participated in another independent measurement before and thus were therefore already familiar with the environment, and were presumably calmer, which could have possibly affected our parameters. By including a variable entitled ‘participated in another measurement’ as covariate in our group comparisons we tried to control for this difference. With regard to the differences in GAs, it must be noted that when we adjusted for our covariates, the results did not remain significant. This may confirm our assumption that women who were in the center longer before the measurement were calmer and thus rated to be less stressed on the pre-rating of the VAS. Of the nine women who had already participated in a study before our measurement, *N* = 7 were in the older GA group and *N* = 2 in the younger GA group. This might explain why women in older GA group felt less stressed at the beginning. Furthermore, almost all participating women had a high school diploma or higher education, which can be seen as a proxy for a higher socio-economic status. Previous studies have shown that women with a lower socio-economic status have a higher stress level per se. This could be due to financial problems, work overload, lack of family support etc. [2, 47–49]. Further studies should therefore include participants with more diverse socio-economic statuses.

On the basis of a study by Doberenz and colleagues [50, 51], SCL and heart rates are known to be lower in sleep than when awake. During the measurement, five women fell asleep (Music: *N* = 2; Guided Imagery: *N* = 2, Resting: *N* = 1), which can lead to a different baseline in SCL and thus to a less significant decrease in SCL compared to women who remained awake for the whole intervention. In addition, we asked all women before each measurement if the room temperature was comfortable, but we did not check this with a thermometer in the room. Nevertheless, we can assume that the temperature was constantly warm over the course of the study (approximately between 22 and 25 degrees (Celsius)). However, since SCL are directly related to environmental temperature, this must be listed under limiting factors of the study. Additionally, we did not follow up the women in later periods of pregnancy to realize if acute relaxation can affect their stress.

## Conclusion

The results of this study suggest that prenatal acute relaxation, even if applied only once, can be a useful non-pharmacological tool to provide a higher state of maternal well-being during pregnancy. Improving maternal wellbeing and reducing stress in this way might mitigate potential negative effects of maternal stress on the fetus.

Despite its limitations, this study was the first of its kind to show that, independent of acute active or acute passive relaxation, a relaxation intervention (*music, guided imagery, resting*) can affect a reduction in maternal stress symptoms in the third trimester of pregnancy.

Interestingly, it seemed that the subjective psychological relaxation effect tends to decrease with GA. Notably, the results of this study show that there are differences between perceived subjective relaxation and the underlying physiological correlates. Despite some women rating the guided imagery as very relaxing, maternal heart rate increased over time in this group.

Further research is clearly required to specify methods of relaxation during pregnancy. Follow-up studies will therefore be necessary, in particular to investigate the long-term effects of relaxation techniques on subjective and physiological stress levels on both the mother and the unborn child.

## Supplementary Information

**Additional file 1: Supplementary Fig. 1**. Maternal Heart Rate (HR): Overview of maternal HR (bpm) over all time points: Music Group, Guided Imagery Group, Resting Group. **Supplementary Fig. 2**. Skin conductance level (SCL): Baseline corrected SCL (microsiemens) over all timepoints for the three groups separately.

## Data Availability

The dataset used and/or analysed during the current study are available from the corresponding author on reasonable request.

## Abbreviations

EDA: Electrodermal Activity
SCL: Skin conductance level
GA: Gestational Age
VAS: Visual Analogue Scale
POMS: Profile of Mood States
TICS: Trier Inventory of Chronic Stress
PDQ: Prenatal Distress Questionnaire
RMSSD: Root mean square of successive differences
HR: Heart Rate
bpm: Beats per minute
SSCS: Short Scale of Chronic Stress
SD: Standard deviation
