# Supplementary material for: Acute relaxation during pregnancy leads to a reduction in maternal electrodermal activity and self-reported stress levels
Source: BMC Pregnancy Childbirth. 2021 Sep 17;21:628. doi: 10.1186/s12884-021-04099-4 (PMC8447712; doi:10.1186/s12884-021-04099-4)
Supplement: Supplementary file 1 — Additional file 1: Supplementary Fig. 1. Maternal Heart Rate (HR): Overview of maternal HR (bpm) over all time points: Music Group, Guided Imagery Group, Resting Group. Supplementary Fig. 2. Skin conductance level (SCL): Baseline corrected SCL (microsiemens) over all timepoints for the three groups separately. [file 12884_2021_4099_MOESM1_ESM.pdf]

Acute relaxation during pregnancy leads to a reduction in maternal electrodermal activity and self-reported stress levels

Ilena Bauer[1], Julia Hartkopf[1], Anna-Karin Wikström[2], Nora K. Schaal[3], Hubert Preissl[1,6], Birgit Derntl[4,5], Franziska Schleger[1]

[1] Institute for Diabetes Research and Metabolic Diseases of the Helmholtz Center Munich/ fMEG Center; German Center for Diabetes Research (DZD e.V.), University of Tuebingen, Tuebingen, Germany
[2] Department of Women's and Children's health, Uppsala University, Sweden
[3] Department of Experimental Psychology, Heinrich-Heine-University, Duesseldorf, Germany
[4] Department of Psychiatry and Psychotherapy at the University of Tuebingen, Tuebingen, Germany
[5] LEAD Graduate School & Research Network, University of Tuebingen, Tuebingen, Germany
[6]Department of Internal Medicine IV, Division of Endocrinology, Diabetology, and Nephrology, University Hospital Tuebingen, Tuebingen, Germany

Correspondence to:
Ilena Bauer
fMEG-Center
Otfried-Müller-Strasse 47
72076 Tuebingen
Email: ilena.bauer@uni-tuebingen.de

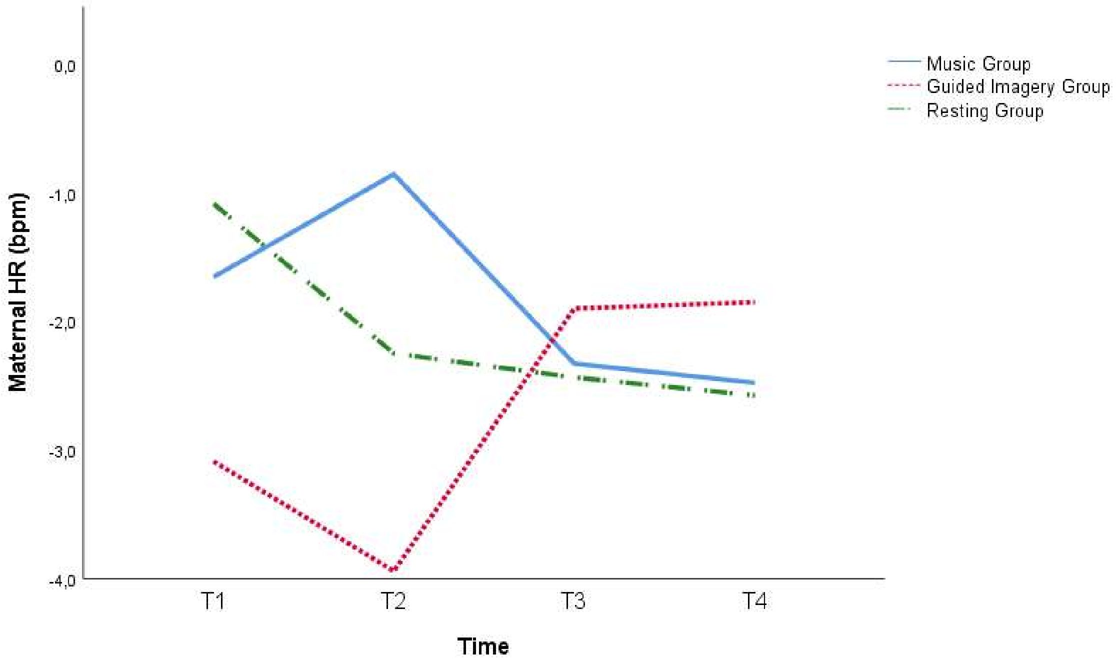

Supplementary Figure 1. Maternal Heart Rate (HR): Overview of maternal HR (bpm) over all time points: Music Group, Guided Imagery Group, Resting Group.

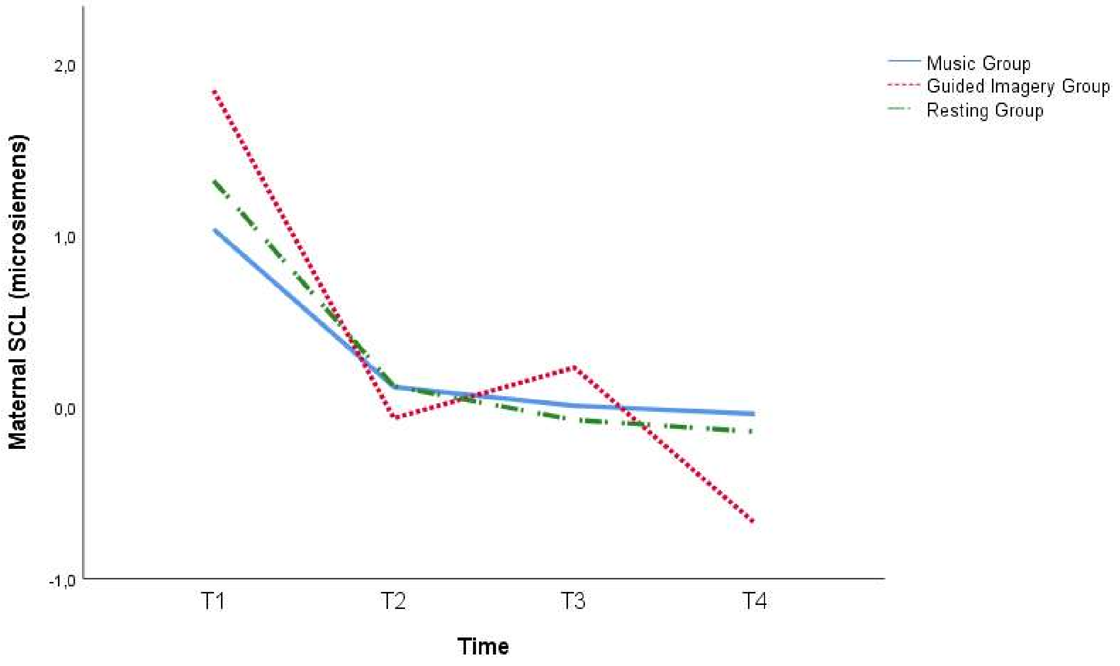

Supplementary Figure 2. Skin conductance level (SCL): Baseline corrected SCL (microsiemens) over all timepoints for the three groups separately.
